# Relevance of Vitamin D and Its Deficiency for the Ovarian Follicle and the Oocyte: An Update

**DOI:** 10.3390/nu14183712

**Published:** 2022-09-09

**Authors:** Arkadiusz Grzeczka, Szymon Graczyk, Agnieszka Skowronska, Mariusz T. Skowronski, Paweł Kordowitzki

**Affiliations:** 1 Department of Preclinical and Basic Sciences, Faculty of Biological and Veterinary Sciences, Nicolaus Copernicus University, Gagarina Street 1, 87-100 Torun, Poland; 2 Department of Human Physiology and Pathophysiology, School of Medicine, Collegium Medicum of the University of Warmia and Mazury, 10-082 Olsztyn, Poland

**Keywords:** vitamin D, oocyte, maturation, embryo, in vitro fertilization, supplementation

## Abstract

For many years, vitamin D (VD) has been known to be an essential micronutrient with important relevance not only for the skeletal system, but also for numerous other mammalian organ systems. Low levels of VD result in a VD deficiency, which is a global health problem. Moreover, VD deficiencies are linked to several pathologies, for instance, diseases of the cardiovascular system, diabetes mellitus, or sub- and infertility. In the past two decades, an increasing body of evidence has shown that adequate physiological levels of VD are crucial for the female gamete and its microenvironment, and VD deficiency has been associated with decreased live birth rates among women undergoing in vitro fertilization (IVF). With regard to the female reproductive tract, VD receptors (VDRs) have been detected in the ovary, endometrium, and the placenta. Although it has been reported that VD seems to be relevant for both calcium-dependent and independent pathways, its relevance for the oocyte’s developmental competence and life span remains elusive. Therefore, herein, we aim to provide an update on the importance of VD and VD deficiency for the oocyte and the follicular microenvironment.

## 1. Introduction

There is no doubt that vitamin D (VD), VD deficiency, and VD supplementation are of important relevance for medicine and public health, since bone fractures, osteoporosis, or low bone mass are a general problem, especially in the elderly. People now tend to reach old age more often, and there has also been a detectable age shift in terms of women’s first pregnancy, which further underlines the importance of the aforementioned fact. In consequence, research over the past two decades did not only concentrate on the importance of VD for the skeletal system and bone mineralization, but also for the female reproductive system. Prior to going into detail on VD’s relevance for the oocyte, some general facts on VD will be provided in this introduction section. To begin with, it is important to know that the main site of VD production is the skin, and the reaction substrate for the synthesis of this secosteroid is 7-dehydro-cholesterol (7-DHC). Ultraviolet (UV) radiation converts it into previtamin D. The isomeric form of previtamin D, cholecalciferol, either reaches the liver unbound or bound to vitamin D-binding protein (DBP) via the blood stream [1]. There, hydroxylation via the enzyme 25-hydroxyvitamin D takes place, and 25-hydroxyvitamin D (25OHD), also known as calcifediol or calcidiol, is generated. The renal enzyme 1α-hydroxylase, encoded by the gene CYP27B1, converts calcidiol to 1,25-dihydroxy-vitamin D (1,25-dihydroxy-cholecalciferol 1,25(OH)2D3), also known as calcitriol. Target cells absorb calcitriol in an unchanged form [2]. The cellular transport of calcitriol is ensured by the activity of heat-shock proteins (Hsp). During this process, the intracellular vitamin D-binding proteins (IDBPs) enhance the vitamin D-binding capacity of Hsp70 [3,4]. It is worth mentioning that the adequate concentration of VD is considered to be crucial for reproductive processes in women [5]. In women of reproductive age, meaning before menopause, a serum 25-hydroxyvitamin D3 (calcifediol) level between 12.0–20.0 ng/mL is considered as VD insufficiency, and concentrations below 12.0 ng/mL as VD deficiency [6,7]. According to the Institute of Medicine, the dietary intake of VD should range between 600 and 800 IU per day, corresponding to a total 25-hydroxyvitamin D level of at least 20 ng/mL. Other societies recommend a dietary intake of VD ranging from 800 to 2000 IU/day for adults 50 years of age or older [8].

**Figure 1:**
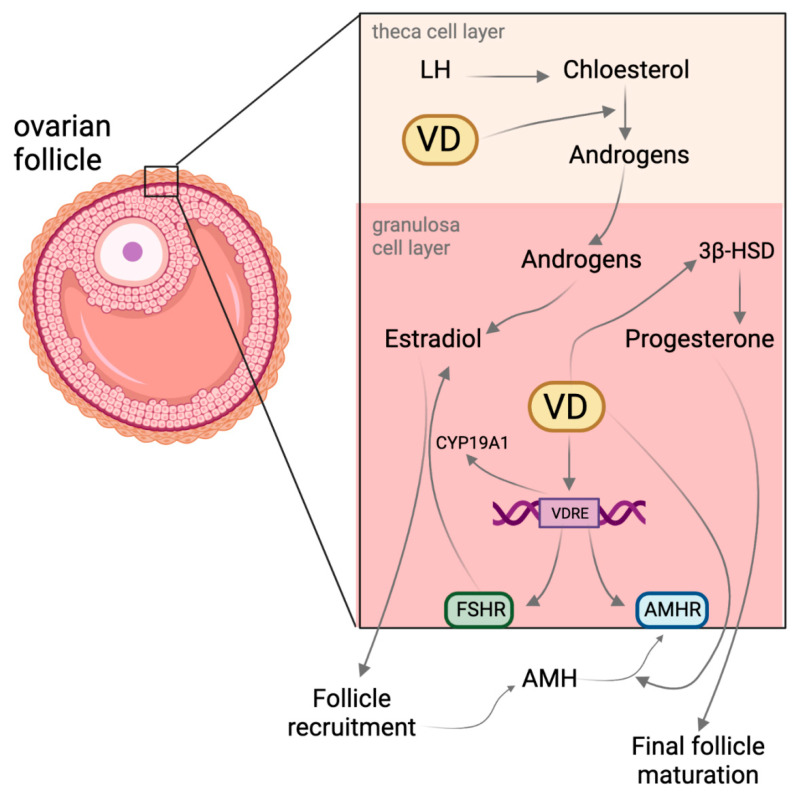
Molecular overview of vitamin D (VD) action in theca cell and granulosa cell layer. VD regulates hormone production and receptor expression in theca and granulosa cells of developing follicles. This, in turn, affects follicle recruitment and maturation. VD also affects 3-β-hydroxysteroid dehydrogenase (3βHSD) and the anti-Müllerian hormone (AMH). Abbreviations: AMHR, anti-Müllerian hormone receptor; FSHR, follicle-stimulating hormone receptor; VDRE, vitamin D response elements.

Interestingly, VD biosynthesis appears to also be possible at the ovarian site [9]. In the ovary, VD has effects on pre-antral and antral follicle development (Figure 1), and 25-hydroxyvitamin D can be detected in the follicular fluid [9]. The fluid microenvironment of antral follicles is regulated by granulosa cells, theca cells, and the transudate secretions of the capillary vessels of the ovary. The composition of the follicular fluid (FF) is strictly defined, and varies with age and cycle phase in women [10,11]. Specific changes in follicular fluid composition create favorable conditions for subsequent stages of female gamete development [12]. One of the dynamically changing systems in the FF is the set of proteins. Variations in their composition ensure functional and structural differentiation of follicles [13]. Sex hormones that modulate the activity of cells surrounding the oocyte are involved in controlling FF composition. This includes, for example, the hormone GnRH, which modifies the gene expression in granulosa cells [14]. Noteworthy is the fact that the VD concentration in FF is higher than in blood serum, but VD is required differently according to the stage of follicular development [15]. However, it cannot be neglected that there is also a bidirectional detrimental role between VD deficiency and metabolic diseases such as obesity, with negative consequences on the ovarian function [16]. Based on recent studies, in the following sections, we aim to shed light on the relevance of VD and VD deficiency for the mammalian ovarian follicle that bears the oocyte. Herein, we will mainly deal with the questions of how VD influences follicle maturation, oocyte quality, and, in consequence, after fertilization, the embryo quality and implantation. At the very end of this article, the interplay between VD and DNA methylation is briefly presented, since epigenetics do play a role, especially during early embryogenesis, and might have important impacts on the offspring.

## 2. Vitamin D and Its Effect on Follicle Maturation

During early folliculogenesis, by far the most important controller of follicle development is the anti-Müllerian hormone (AMH) [17,18,19]. AMH is the so-called ‘guardian’ of ovarian reserve, i.e., it prevents the excessive activation of primary follicles, and the development of too many antral follicles by reducing their receptor sensitivity to folliculotropic hormone (FSH) [19]. Interestingly, VD-responsive sites were demonstrated in the AMH coding sequence [20]. AMH is downregulated under the influence of VDR (Figure 1), but a synergistic effect of AMH and VDR seems possible, too [21]. Previous research on women undergoing ovarian stimulation for IVF has shown that there was a twofold increase in AMH receptor II (AMHR-II) expression in the granulosa cells of women with insufficient follicular fluid calcitriol levels (<30 ng/mL) compared to women with normal levels (>30 ng/mL) [22]. This indicates a reciprocal regulation of the two hormones. Interestingly, a positive correlation between AMH and VD was detected in women with fertility disorders such as polycystic ovary syndrome (PCOS), primary infertility, or established infertility [23,24,25]. In consequence, it appears likely that VD is able to promote oocyte activation and maturation via the downregulation of AMHR-II.

Moreover, VD signaling leads to an increased production of steroid hormones in granulosa cells (Figure 1) [26], which are crucial for the oocyte maturation and pregnancy, too. An increase in progesterone production upon vitamin D treatment has been reported [22]. Vitamin D and progesterone share structural and functional characteristics and also exert similar effects [27]. Vitamin D is able to upregulate, to a limited extent, the expression of proteins associated with progesterone metabolism (Figure 1) [22,28]. The increased production of 3β-hydroxysteroid dehydrogenase (3β-HSD) mRNA following vitamin D treatment is an example of the regulatory capabilities of the VDR [22].

## 3. Vitamin D and Its Effect on Oocyte and Embryo Quality

Adequate levels of calcitriol seem to be necessary to regulate key processes during development, differentiation, and genomic imprinting through epigenetic mechanisms in oocytes. VD and its clinical implications regarding the developmental competence and fertilization of oocytes has prompted researchers’ attention. As previously mentioned, VD deficiencies in both serum and follicular fluid may lead to fertility disorders [29]. Due to ethical restrictions on human oocytes for experimental reasons, animal models related to VD deficiency have been established and widely used to investigate the impact of maternal deficits on oocytes, embryos, and the offspring. In particular, the murine species is commonly used for investigations into the effects on offspring. However, one has to keep in mind the limitations regarding species differences, and the different reproductive physiology and life span between the murine and the human species. Consequently, the summarized results generated in animal models have to be treated with caution, and should not be extrapolated one by one to women. Nevertheless, the data from mouse models can be obtained faster, and more invasive manipulations with regard to the ovary and oocytes are possible, and do provide a first hint or trend for further research. A recent mouse model study revealed that in the case of VD deficiency and hypocalcemia at the same time, a significant reduction in oocyte retrieval after ovarian stimulation was observed, and the generated oocytes showed a poor maturation ability [30]. This negative effect could be reversed after normalizing serum calcium levels in the oocyte donors. This link between VD and calcium is of special importance for the mammalian oocyte, since Ca^2+^ oscillations are required for crucial processes such as the resumption of meiosis, polyspermy block, male chromatin decondensation, recruitment of maternal mRNAs, and pronuclear formation [31]. Interestingly, aged oocytes have been shown to present lower amounts of Ca^2+^ stored in the ER, altered cytoplasmic Ca^2+^ uptake due to decreased sarcoplasmic reticulum Ca^2+^ ATPases (SERCA2) expression, and diminished ATP availability secondary to altered mitochondrial functionality [31]. Moreover, the VD level in FF is associated with embryo quality, normal fertilization, and implantation rates, since physiological VD levels are correlated with high-quality oocytes [29].

**Figure 2:**
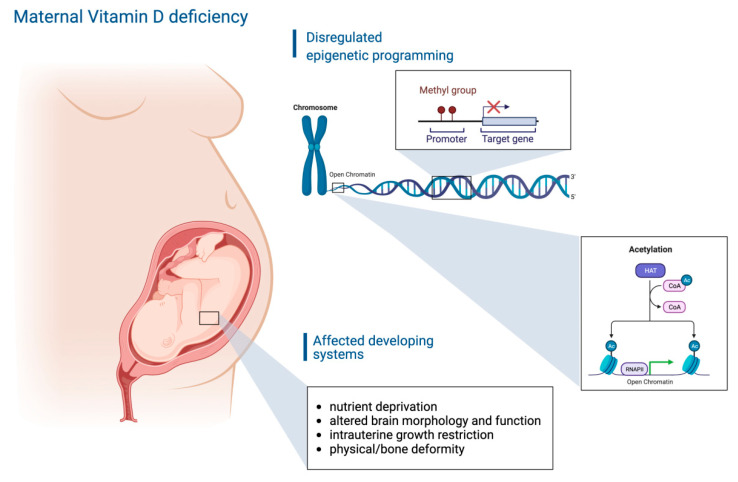
Scheme showing the outcome of maternal vitamin D deficiency on the developing fetus. Based on a dysregulated epigenetic reprogramming and VD deficiency, several organ systems of the offspring can be affected [48].

In addition, it was recently indicated that there is a significantly higher level of follicular fluid VD in women with a lowered ovarian reserve when compared to women with a normal ovarian reserve [32]. This may shed new light on the relevance of VD on female reproductive aging. Ciepiela et al. (2018) have shown that in women with a mean age of 34.3 ± 3.9, a pregnancy after IVF was established in patients with significantly lower vitamin D levels compared to those in which pregnancy was not established (Figure 2) [15]. Follicular fluid vitamin D concentrations higher than 30 ng/mL were associated with 44.4% of women who became pregnant. On the contrary, in 84.6% of women who had a VD concentration of less than 10 ng/mL FF, embryonic implantation did not occur [33].

VD receptors (VDRs) are present in many tissues and have numerous functions in specific organs all over the body [34]. With regard to the female reproductive system, VDRs have been detected in human granulosa cells and in primate oocytes and granulosa cells [9,28]. In somatic cells, receptors for 1,25(OH)2D3 are located in the cell membrane and in the nucleus [35]. The activation of the nuclear receptor leads to genomic and, to some extent, non-genomic responses [36]. The receptor–ligand complex binds to vitamin D-responsive elements (VDREs) in the cell nucleus. A VDRE (Figure 1) is a specific nucleotide sequence in the promoter regions of genes regulated by VD. In addition, VDR can interact with other nuclear proteins, thus modifying the transcriptional activity of the cell more broadly by altering the activities of RNA polymerase II [37,38].

Interestingly, it has been indicated that VD may regulate the degree of cytosine methylation in the promoter region of vitamin D-regulated genes (Figure 2) via interactions with methylcytosine dioxygenases [39]. However, the VD-regulated demethylation rate varies, and the consequences or outcomes might not be apparent until several cell cycles have been completed [40]. Another interesting fact is that VD supplementation seems to slow down the aging process, as recently identified in a DNA methylation study using the so-called epigenetic clock [41]. Moreover, histone acetyltransferases (HATs) or histone deacetylases (HDACs) are VDR partner proteins [42], and HATs facilitate the acetyl group transfer from the acetyl-coenzyme A (Acetyl-CoA) moiety to the NH^3+^ group on lysine. Consequently, modifications within histones modulate the euchromatin density. Other auxiliary proteins may also interact with the VDR. One of these proteins is the nuclear receptor co-repressor 2 (NCOR2), which recruits DNA deacetylases to the promoter site [43]. A similar action mode to that described above can be seen at the nuclear receptor co-repressor 1 (NCOR1), whereas coactivator receptor 2 (NCOA-2) assists nuclear receptors in transporting acetyltransferases to promoter sites [44]. The SNW domain-containing protein 1 is a coactivator that can bind to the ligand-binding domain of the VDR and to retinoid receptors to increase VD and retinoic acid-induced gene expression [45]. Interestingly, as shown in an animal model, the absence of functional VDRs impaired folliculogenesis drastically and led to uterine hypoplasia [46]. Deactivation of the Cyp27B1 gene, encoding for 1α-hydroxylase, led to the shrinkage of the entire ovary, impaired folliculogenesis, and impaired steroidogenesis [47].

## 4. Conclusions

Vitamin D insufficiency or deficiency and its association with sub- or infertility is a difficult challenge for numerous clinicians and reproductive medicine specialists. Therefore, the safe and ethical modulation of VD and oocyte quality requires a more comprehensive understanding of their interplay. As presented in this article, VD appears to not only have stimulatory effects on folliculogenesis, but also a negative impact on oocyte maturation, based on the adequate concentration and supplementation, respectively. Therefore, VD supplementation in women prior to or during pregnancy is still controversial, since further elucidation is required to answer the question of the extent to which these antagonistic effects are related to individual patients, genetics, or epigenetics. There is no doubt that optimal serum and follicular fluid levels of VD are of high relevance for female reproduction, with the oocyte at the top. Importantly, the establishment of accessible and non-invasive tests in which VD can be used as a biomarker for oocyte quality would be especially helpful to determine which therapeutic strategy is best suited to individual clinical cases. Taken together, elucidating the molecular mechanisms and the unique role of VD for females and for their oocytes would be a solid basis to identify therapeutic targets to improve oocyte health. Keeping in mind the negative associations also related to VD supplementation, appropriate concentrations of VD could be set for specific patients as a new strategy to enhance and prolong female reproductive fitness.
